# Women’s experiences with non-invasive prenatal testing in Switzerland: a qualitative analysis

**DOI:** 10.1186/s12910-023-00964-3

**Published:** 2023-10-23

**Authors:** Mirriam Tyebally Fang, Federico Germani, Giovanni Spitale, Sebastian Wäscher, Ladina Kunz, Nikola Biller-Andorno

**Affiliations:** Institute of Biomedical Ethics and History of Medicine, University of Zurich, Winterthurerstrasse 30, 8006 Zurich, Switzerland

**Keywords:** Prenatal testing, Pregnancy, Birth, NIPT, Healthcare

## Abstract

**Background:**

Prenatal genetic testing, in particular non-invasive prenatal testing (NIPT), as well as screening for risks associated with pregnancy, and counseling, play pivotal roles in reproductive healthcare, offering valuable information about the health of the fetus to expectant parents. This study aims to delve into the perspectives and experiences of women considering genetic testing and screening during pregnancy, focusing on their decision-making processes and the implications for informed consent.

**Methods:**

A nationwide qualitative study was conducted in Switzerland, involving in-depth interviews with women who were 1 to 2 years post-partum, covered by basic compulsory Swiss insurance, including women with a migration background. Thematic analysis was employed to identify key themes and patterns in the data.

**Results:**

The findings underscore the significance of effective communication during prenatal counseling, suggesting that healthcare providers could not only convey technical information but also support women in their decision-making processes. Women need comprehensive information about genetic testing and its implications, as well as the reasons for screening during pregnancy, as there might be a need to bridge knowledge gaps and clarify misconceptions. Furthermore, the study highlights the multifaceted nature of decision-making, with women considering factors such as uncertainty, values, emotional responses, and societal support systems. The concept of acceptance emerged as a crucial theme, with some women expressing their readiness to love and accept their child, regardless of genetic anomalies or disabilities.

**Conclusion:**

This study offers valuable insights into the perspectives and needs of women regarding prenatal genetic testing, screening, and counseling in Switzerland. It underscores the importance of enhancing the clinical interaction and informed consent process by providing comprehensive information, addressing misconceptions, and supporting women in decision-making about pregnancy management and the management of the child’s health, following prenatal genetic testing, including NIPT. These findings can inform healthcare providers and policymakers in improving the quality of prenatal counseling, ensuring informed consent, and supporting women in making well-informed and meaningful decisions about genetic testing, and on the use of screening during pregnancy.

**Supplementary Information:**

The online version contains supplementary material available at 10.1186/s12910-023-00964-3.

## Introduction

Pregnancy has been posited to be an epistemically transformative experience [1, 2], in that it is the experience of pregnancy that allows a pregnant woman to ‘cognitively entertain certain content’, understand things in a new way and gain new information that would otherwise be unavailable to her without this experience [3]. As a result of the transformative aspect of the pregnancy experience, a woman may find that her values and preferences may not always be stable and predictable, and some women may find it challenging to anticipate and take a clear position when faced with a decision [3].

Despite the possibly complex psychological shift some women might experience, they are nonetheless required to navigate the processes associated with pregnancy from the position of their present-day selves. In terms of the medical experience of pregnancy, women need to have a broad understanding of both the medical and non-medical issues encompassing psychological and medical health issues such as pregnancy-induced diseases, foetal development, the birthing process, and post-partum recovery.

Medical decision-making is challenging as patients are presented with often complex information, unfamiliar medical terminology and the need to interpret and apply statistical data to their individual situation. On top of this, many medical decisions in pregnancy present additional ethical challenges for women. One such challenging decision is to undergo prenatal testing for chromosomal abnormalities, since these may be associated with miscarriage [4] and might lead women to have to consider terminating a pregnancy.

Non-invasive prenatal testing (NIPT) is a development in prenatal testing, in which fragments of the fetal genome obtained from the maternal circulation are analyzed for fetal abnormalities, with a high specificity and sensitivity [5–7]. NIPT allows women to screen for certain aneuploidies, such as trisomy 21, 18, and 13 [8], potentially saving them from more invasive prenatal tests which carry the risk of miscarriage [7, 9–11]. The test is offered to women as a means to expand their autonomy in the context of their pregnancy, by giving them the choice to evaluate the ‘health’ of their fetus, with the benefit of testing while reducing risks for the health of the fetus, such as for the cases of amniocentesis and chorionic villus sampling [11]. The test is presently commercially available to all pregnant women, irrespective of their risk profile.

The choice to undergo NIPT encompasses the spectrum of challenges in pregnancy decision-making. For instance, pregnant women must decide the degree of medical involvement in the management of their pregnancies, have to evaluate and understand the significance of the functioning and results of such tests and their limitations, including the conditions being tested for, contextualizing the impact on their present situation, their future self as a potential parent, and that of their potential future-child, and decide on the follow-up actions of invasive testing and whether or not to consciously continue with the pregnancy.

Empirical research on NIPT has frequently focused on practical and procedure-based considerations, aiming to guide its implementation within health systems [12–15]. This included women’s values on test safety [14–24], test precision [14–19, 22, 23], procedural ease [14, 15, 20, 21, 24], test timing in relation to the gestational age of the pregnancy [11–16, 19, 22–26], and their view on NIPT compared to other forms of prenatal testing or screening [14, 15, 17, 23]. Other issues included financial considerations [15, 18, 20, 23, 24, 26], counselling for NIPT and measures of informed consent [13, 14, 18, 21–27]. Given the complexity of prenatal testing decisions with NIPT, more attention has been directed towards elucidating the decision-making process pregnant women undergo in NIPT-related decisions [20, 28–32]. Previous research recognizes that defining counseling strategies and informed consent best practices is a demanding task. Understanding how women construct their understanding of genetics, their concerns, their reasoning and considerations, as well as the influential factors in decision-making, could allow medical practitioners to better support pregnant women taking these important decisions. Furthermore, gathering this knowledge would suggest avenues through which medical practitioners could achieve the goal of improving decision quality among pregnant women in prenatal testing and the use of screening [33–35].

In Switzerland, prenatal genetic testing is widely available for expecting mothers since 2012 [36], and since 2015 it is covered by health insurance companies for women at intermediate or high risk after first trimester screening. This test serves the purpose of estimating the likelihood that the fetus may possess a genetic anomaly, such as Trisomy 21 (commonly known as Down syndrome). This probability calculation relies on the interplay of three factors: age, specific markers detected in the mother’s blood, and the measurement of nuchal translucency via ultrasound, typically performed between the 11th and 14th week of pregnancy. This policy led to a sharp increase in NIPT uptake in Switzerland [37]. According to Swiss law, abortion is legal based on the judgment of a physician who must assess the risk of serious physical injuries and/or psychological distress, and must be carried out within twelve weeks of pregnancy initiation [38]. Termination of pregnancy is a medical procedure that must be covered by basic health insurance [39]. In the following, we explore how women in Switzerland (including women with migration background) faced with a decision to undergo prenatal testing with NIPT or screening construct their understanding of NIPT and genetic variations, recognize their evolving identities and corresponding shifts in values, the factors influencing their decisions on screening and/or prenatal testing, and how they cope with abstract and uncertain information, such as the likelihood that their newborn child will develop conditions due to the presence of genetic abnormalities.

## Methodology

### Inclusion criteria, study recruitment and structure of the interview

We designed a qualitative study using narrative inquiry according to the DIPEx methodology [40–43] to explore women’s pregnancy experiences. All women who were between 1 to 2 years post-partum and covered by the compulsory basic insurance in Switzerland were eligible to take part in the study. The demographics of participants recruited in our study, including but not limited to age, education, whether they accepted, rejected, or were doubtful about NIPTs, and their nationality, are described in Supplementary file 1. This included women who had miscarried, had stillbirths, and who had chosen to terminate their pregnancy. The decision to wait 1-year post-partum was to allow women time to reflect on their pregnancy and birth experiences, and the decisions they made during this time. They were purposefully recruited such that the sum of demographic, social and medical factors would fulfil maximum variation sampling criteria. Participants were referred to the study team from across the country through the public hospital system, community-based midwifery and paediatric-nursing networks across the country, a non-governmental organization supporting mothers who have lost a pregnancy (including by choice), playgroups, and social networks. In total, 37 women, 26 of whom were Swiss citizens, and 11 of whom were migrants to Switzerland on residence permits, were recruited between January 2018 and December 2019. Interviews were carried out in the preferred language of the participant. In total, 23 interviews were carried out in English, 10 in German, 3 in Italian and 1 in French. Informed consent was taken prior to commencement of the interview. Given the sensitive nature of the topic, participants were reassured that they could choose to skip a question or stop the interview at any time. The interview would begin with a broad, open-ended question: “Could you please tell me the story of your pregnancy?”. Participants were given full freedom to respond to this question without being interrupted by the interviewer. This was then followed by a semi-structured interview on the themes of parental responsibility, values, and community support; prenatal visits, including experiences with and knowledge of prenatal testing and screening; and the process of decision-making about prenatal testing and screening. The interview guide is available as a supplementary file (Supplementary file 2).

### Data security, coding and analysis

The interview data was given a code, and uploaded onto a secure server hosted by our research institute, with access provided exclusively to the research team. All interviews were transcribed verbatim. Identifying information was anonymized. The initial coding structure was developed coding interviews in English, German, French, and Italian by native speakers in our team (MTF, GS, SW, LK, CMD). Once the coding tree was consolidated, German, French, and Italian transcripts were securely translated into English using the automated translation service DeepL Pro so that all researchers could understand their contents, although further processing of the data was done in the original language of the interview by our multilingual team. No interview data was stored by DeepL Pro. The finalized transcripts in their original language were uploaded into MAXQDA and coded. The coded data was then thematically analysed. The entire methodological process has been described by Spitale et al. 2023 [44].

## Results

### Communication with Medical Staff

In this section, we focus on communication with medical staff, including physicians, OBGYNs, nurses, and genetic counselors, in relation to genetic testing specifically. For most interviewees, this referred to NIPT, although other prenatal genetic tests and other prenatal tests mentioned in association are included here. In general, counselling seemed to focus on the procedural and administrative aspects of testing. Almost all participants were informed of the relative costs of NIPT compared to the ‘standard’ first trimester screening tests, and were told their eligibility for insurance coverage based on their risk profile. Very few participants were counselled about the qualitative test findings, such as the conditions tested for, and were mostly given brochures to read in their own time. While some felt they could then clarify the set of information provided in the brochures with their doctors, others felt their doctors were not able to adequately answer their questions.

*She announced one examination before that she will ask me about that (NIPT), and she gave me a brochure. She said read that, and then when you come next time we make it (the test) or we don’t make it.*

*PPT36_E23S, 01:10:41 – 01:11:00*

Some mentioned that they did not feel guided by their doctors to think about what to test for, the potential consequences, and possible follow-up actions. Some participants were misinformed about the certainty of NIPT compared to other screening tests, perceiving it as certain from a diagnostic perspective. Some participants were also misinformed about the range of conditions that NIPT could screen for. For example, a mother who fell unexpectedly pregnant while on warfarin, a drug typically used to prevent blood clots from forming that can lead to miscarriage or birth defects, and was concerned about its teratogenic effects chose to do NIPT to reassure herself, even though it would not have been possible to test for these using NIPT.

*I was even on quite heavy blood thinning medication at the time and I had been told repeatedly not to get pregnant on those. One morning I woke up and I thought “Weird, I think I have pregnancy symptoms,” after three times you kind of know. (…) I remember the phone call coming (NIPT results) that everything was okay and confirming the sex of the baby. There was a massive release—relief for everybody.*

*PPT05_E5S, 00:11:52 – 00:12:07; 00:13:50 – 00:13:59*

Not all participants were informed about NIPT by their doctors. Some managed to independently source marketing material for NIPT, which they then brought to their doctor’s office. Many felt their initiative and insistence on testing made them appear sufficiently informed and educated about genetics and NIPT. As a result, their doctors did not provide further counselling, raising issues of professional duties in informed consent.

*Interviewer: How were you counseled about NIPT?*

*Interviewee: I wasn't at all. No, I just counseled myself.*

*Interviewer: Ah, okay. And then you- did you tell the doctor that I want to do this? Or did she actually mention it to you?*

*Interviewee: No, no, I told him.*

*Interviewer: Aha, and how did he respond?*

*Interviewee: (laughs) He um, um, he was fine, my doctor was fine with everything. Um, I actually brought him the [commercial name of the test], like marketing sheet and I said, "Okay, I want to do this test".*

*PPT22_E12I, 00:38:39 – 00:39:13*

*Interviewer: Did-did your doctor tell you about the – I mean I know that you initiated it and you told him most things that you wanted to do but did he actually tell you again what are the benefits, or the harms [of the test]?*

*Interviewee: No, no, he just said, "Okay, if you want, it's fine and we'll go tweak the measurement.". So he was– And I think, I started the discussion because he could- before he could even do it.*

*Interviewer: Yeah. And then did he tell you about making future decisions. Like, "If you do this then we might have to think about sending you to a genetic counselor or you might have to think about termination or whether not you wanna keep the child." Was that something that he spoke about?*

*Interviewee: No.*

*PPT22_E12I, 01:23:25 – 01:24:07*

Others did not realize NIPT was an option at all, despite raising personal concerns about inheritable physical and mental conditions. One such interviewee only realized upon reflection post-pregnancy that there were alternatives to the ‘standard’ routes of testing she was offered that could have answered her real concerns based on her family history (see PPT12_E8I, 01:02:18 – 01:03:13 below). Another, whose child had shown physical deformities on prenatal ultrasound, was told by her doctor that even if they did find chromosomal abnormalities, there was ‘no doctor in the world’ who could help interpret this information in relation to the life, health and wellbeing of their child, and that all people could have abnormalities they were unaware of, and live ‘perfectly fine’ with it (PPT32_E21S, 00:32:00—00:33:09). Many also felt they could have been better supported emotionally in discussions about prenatal testing.

*I think some emotional support there would actually be very sensible, and obviously in particular if there's any type of risk.* ***(…)*** *I think starting a conversation (…) about how you feel about it, or what are your thoughts on/ maybe understanding the patient's needs more than assuming, the standard options, which you can always fall back to, you can say, look, this is the standard option. I mean, honestly, I wasn't even aware of what other options there are.*

*PPT12_E8I, (family history of mental and physical disability) 01:02:18 – 01:03:13*

Emotional support is also important as pregnant women may struggle with insecurities about the health of their pregnancy and fetus, making them vulnerable to agreeing to tests they might not want or need. Two participants in particular were notably vulnerable compared to the rest. They were informed of being at high risk for trisomies despite explicitly stating that they did not wish to do any prenatal screening, but felt subsequently compelled to follow-through with more extensive testing, including invasive tests. They were not aware that the nuchal translucency of their fetus was being measured while undergoing an ultrasound, and did not question that an ultrasound was performed as these are seen to be done routinely in pregnancy. They mention the importance of determining and respecting the limits of screening, and emphasize that nuchal translucency tests should not be done if someone does not want prenatal screening.

*In retrospect, we felt a bit caught off guard, because at first we didn't want to do anything and then we realized, ah, now we've seen it anyway. Yes, I think it's a pity that doctors don't point out more what the consequences of every test, of every examination you do are. That one should also consider if someone doesn't want to do this first trimester test, then we don't do a measurement of the nuchal fold.*

*PPT34_G8S, 00:41:23 – 00:42:01*

The concept of ‘risk’ was often used to direct conversations about NIPT. Many interviewees struggled with understanding risk based on numbers and ratios. For some, a low risk, such as 1 in 20,000 chance of a genetic abnormality, was still perceived to be an issue, as they felt they could be the unlucky ‘1’. Others flipped the numbers around so that the numbers reflected having a child without a genetic abnormality. In this way, even a ‘high’ risk such as a 1 in 10 chance of a genetic abnormality was reassuring, in that 9 out of those 10 children would be born without the conditions screened for (PPT18_G6S, 00:31:33 – 00:32:37).

*She said 1:2 or something, 10,000: 20,000. I suppose it was a good ratio. A better ratio would have been 1:10 or 1:100.*

*PPT30_E19S, 01:14:05 – 01:14:14*

Risk was also used as a gatekeeper to test access for some women. Although the test could potentially be paid for out of pocket on the request of the patient, some women were directed by their doctors to follow the standard protocol, with additional tests entertained only if the ‘risk’ deemed it necessary.

*Right from the start I said, “Look, you know, we both have disabled people in our families, we both know what this means for our family and that it can be a huge thing, and I really want to know as much as I can, whether there's any sign of a disability or not.” The doctor didn't enter into that conversation much because he just said, “Well, you know, we'll still do the standard tests and see what happens.”*

*PPT12_E8I, 00:14:23 – 00:14:50*

### Knowledge of genetic abnormalities

Among respondents with knowledge of genetic abnormalities, the most useful information was derived from reflecting on personal experiences or the experiences of others. In general, there was a divide between Down’s Syndrome, that was unanimously seen as positive in terms of character and quality of life, even though most respondents also recognized that the severity of Down’s Syndrome existed on a spectrum, and ‘other’ genetic syndromes or disabilities – which were thought to be mostly a cause of multi-organ dysfunction (PPT19_E9S, 00:14:30 – 00:14:59), suffering, and overall a poor quality of (short-lived) life (PPT05_E5S, 00:38:44 – 00:39:50). People with Down’s syndrome were described as genuine, warm, sociable, and happy – perhaps even more so than a person without trisomy 21.

*I know that Down’s syndrome touches a bit the brain, I don’t have the details, but I know that they are people who probably live in their world, they are happy in their world as they are and many times they are marginalized when they could be better people than others who don’t have such problems. (…) All I can tell you is that I saw them happy, happy and serene.*

*PPT15_13S; 00:54:34 – 00:56:04*

In spite of this positive impression, many were wary of the care-giving tasks required of them, seeing the dependency of a person with Down’s Syndrome as ‘imprisoning’ and concerned about the future when the parents are old or no longer around. They also expressed concern of the cruelty of the world towards these very amiable people (PPT15_13S, 00:51:52 – 00:53:30).

Disabilities and genetically inheritable conditions other than Down’s Syndrome were less well known and understood. The most commonly mentioned trisomy that concerned respondents was Trisomy 18. Although trisomy 18 was a concern, one participant mentioned that she would not test for it because of the rarity of the condition, despite intending to terminate a pregnancy if the child had Trisomy 18.

*Trisomy 18 or something is so rare that for me that’s not a reason to do the testing because it’s too rare. That’s an important thing for me. Trisomy 18; I would have done an abortion in all cases, but it’s so rare that there is no sense for testing in my eyes.*

*PPT31_E20S; 00:59:24 – 00:59:48*

While most information about Down’s Syndrome was experiential, knowledge about other conditions were mostly obtained through the doctor’s office. Respondents mention receiving a booklet, or being given advice to take supplements from their healthcare providers. In spite of the information received, they do not seem to know much about any of these conditions.

One participant who found she had a translocation that predisposed her to conceiving children with trisomy 13 described how her own research on scholarly websites and her interaction with social media helped her source, understand and contextualize the information she needed. Doctors were not able to adequately respond to her concerns. Other sources of information mentioned by participants included stories, internet resources and the media (via television/youtube).

*Interviewer: Where did you get the information from?*

*Interviewee: Google Scholar pretty much. Just looking at various articles, academic articles. In French, there is Orphanet which is all the rare diseases and they have a little section on Trisomy 13. Something else that really helped for the second pregnancy was Facebook groups. There’s a dedicated Robertsonian translocation Facebook group, and there’s one on Trisomy- like on the 13, 14 specifically. That is like a wealth of information. Part of it is anecdotal, but if you pull all of it together, you do get a lot. Some people were working directly with experts, they were sharing their information as well, that was also very, very helpful.*

*PPT29_E18I, 00:25:41 – 00:26:39*

Most respondents rationalized that humanity existed on a spectrum, with no authoritative definition of ‘normal’. There was recognition that complications not prenatally screened for may arise later in life (PPT05_76), and that we ourselves may have undetected genetic issues.

*We all have difficulties sometimes we don’t even know, unless we actually get ourselves tested. So for me, it doesn’t bother me. It’s just means that I have to, get to know my child in a different way, and accept them for who they are, and find the ways to best help them.*

*PPT05_E5S, 00:31:29 – 00:33:01*

Some respondents recognize they were unprepared for life with a person with a disability. This view was sometimes changed by interactions with people with disabilities, which contextualized the theoretical information that a participant might have had.

*In honesty, nothing at all, and between having my first child and second child, I met up with friend’s of mine, whose sister has Down syndrome, who I haven't seen, the sister, between when she was a child and an adult, and I was quite surprised – this is so awful, it’s so prejudiced, but I was really surprised how we could have a really good conversation.*

*PPT10_E7IS, 73–77; 00:12:21 -00:12:49*

#### Uncertainty concerning prenatal genetic testing, screening, and dealing with uncertainty

The topic of prenatal testing and screening was fraught with uncertainty, affecting participant confidence in decision-making about pregnancy management and fetal health. Participants contemplated a range of scenarios they described as hypothetical, with many describing carefully considered positions on complex topics, followed by acknowledging their uncertainty when confronted by an actual decision:

*“I don't know what we would have (done), and I don't think you can necessarily know till you go through it.”*

*PPT20_E10I, 00:28:33 – 00:28:43*

Topics described as hypothetical included pregnancy progression, prenatal testing results, abortion decisions, emotional reactions to an abortion, life with a disabled child, quality of life of a disabled child, attachment to their prospective child, and support from their social environment.

According to our interpretation, the degree of uncertainty expressed by participants corresponded to a sense of abstractness, and directly impacted their ability to make meaningful decisions. Some participants counteracted this by contextualizing the medical information received. This often happened incidentally through chance experiences with people with disabilities, and sometimes intentionally, through researching and connecting with others in similar situations. Apart from understanding health issues from a medical perspective, access to patient narratives helped parents understand how the lives of others were impacted by these issues, coping strategies, potential outcomes, and the available social, financial and medical infrastructure. Participants recognized the emotional aspect of these decisions was not always predictable. Many took it upon themselves to make a decision beforehand, so as not to be affected by their emotions*.* A handful of participants recognized shifting values over time, affecting their decisions. This most often happened after life-changing events, such as the birth or loss of a child. Some mentioned they not want to re-live the pain of losing a child, even if the new child they carry had genetic anomalies (PPT35_G8S, 00:34:05 –00:34:20). Others recognized the connection between a foetus and a child after giving birth and were inclined to continue a pregnancy regardless of the health or genetic status of the prospective child, even if they felt differently before.

*Before I got pregnant, we both said that if a test indicated a very high probability of the child being disabled, or if we’d known for sure even that the child was disabled through scans or whatever, we would have probably opted for an abortion.*

*We were both pretty clear on that impact on us. We knew about how big this type of impact can be, and we, at least at that stage, were not willing to go for that. I must say interestingly both of us, on the second pregnancy, were less clear on, on that choice.**** (…)****Having had the first child, and gone through the process and having this you know cute boy, I mean, you know, this embryo will become a child, right? A disabled child or a healthy child or something, but the link between the life, the later life and the embryo is stronger once you've seen it once. And choosing to end that link is a harder choice then. We then actually pre-decided not to decide, so we then decided on the second pregnancy that, we would decide if we got into the situation where a test indicated a high probability of some disability.*

*PPT12_E8I, 00:12:18 – 00:13:34*

Participants who struggled with decision-making under conditions of uncertainty found various cope strategies. One strategy was boundary setting, for example by limiting prenatal testing or screening, limiting visits to their gynaecologist, and being clear of the purpose of these visits.

*We also knew, or rather we had also decided against these tests, because we were of the opinion that we would be able to lead a good life, a fulfilled life, even with a restricted child.*

*PPT34_G8S, 00:53:30 – 00:53:46*

A second way was by deferring to an external authority. Participants generally trusted their doctor’s competence, and assumed that the authorities considered morality as part of their guidance. In our interpretation of the data, participants following medical advice therefore interpreted standardized prenatal tests as acceptable and ethical. A small number of participants however were able to recognize that medical opinions differed among different professionals, and were neither always neutral nor ‘correct’.

*I think I know better now what I want. I feel like I could be more assertive with medical staff. Because I now know that not everything they say is true. You hear so many different opinions. One nurse tells me this, and another one tells me that.*

*PPT07_G1S, 01:22:38 – 01:22:54*

#### Acceptance

Many participants mentioned the need for acceptance. The concept of acceptance was applied in different ways. For some, this meant the acceptance of differences and supporting the development of an inclusive society (PPT30_E19S, 01:11:10 – 01:12:00). For others, acceptance referred to letting go of the sense of control and security prenatal testing provided, realizing not only that prenatal testing was not comprehensive, but also that a multitude of non-genetic issues could complicate the pregnancy and the health of their child. Lastly, almost all participants mentioned they were likely to love and accept their child at birth, regardless of its health or genetic anomalies. Interestingly, almost half of interviewees underwent NIPT screening, suggesting that their predisposition to accept and love a child with special needs is not dependent on expecting mothers’ willingness to undergo NIPT testing.

*My mind would have wandered to all these questions which are totally unnecessary to ask yourself because it's such an abstract situation, and once the child is here, it's your child and you feel about, you feel so much love.*

*PPT19_E9S, 00:50:33 – 00:50:54*

At the same time, some still felt a sense of responsibility to prenatal testing and screening, mentioning the guilt they would feel if they chose not to test and the pressure they faced by the medical system towards testing and ‘normality’.

*If it's something that could have been detected, for example, if I had refused to have scans, and then boom, something happens, then I would feel guilty.*

*PPT29_E18I, 13; 02:08:19 – 02:08:25*

Parents were also concerned about the lack of social support, based on their observations and discussions with others in similar situations (PPT27_E17S; 01:25:55—01:26:07; 01:27:27 – 01:28:38). Most of those who shared this view considered termination of pregnancy a possible follow-up action, with one drawing a parallel between abortion and euthanasia (PPT34_G8S, 01:35:37 – 01:36:25). Other participants compared life with ‘normal’ children to life with a potentially disabled child. They acknowledge that life is changed drastically by the addition of any child, and so sees the change as a matter of expectation and adaptability to the specific child.

*For us it was clear that if the child had had trisomy 21, then this would stay with us, then we would find a way that satisfied us all. We are also ready to change our lives and change does not have to mean that it becomes bad. It can also be, or it will also be, a change for the better. You would just have to give up certain things, but you have to do that anyway.*

*PPT34_G8S, 00:53:52 – 00:54:16*

## Discussion

The medical community has made efforts to align genetic testing and prenatal screening with the parental values, primarily through careful counselling and the shared decision-making approach [45–49]. Our findings provide valuable insight into improving this process by addressing the needs of women contemplating NIPT. It also offers the opportunity to clarify the professional duties of gynaecologists counselling women.

One aspect of professional responsibility that needs to be emphasized is differentiating standardizing information provision from standardizing prenatal care. In the experience of women interviewed, doctors deferred to insurance coverage and ‘standard’ testing as gate-keepers to further prenatal tests. Health providers need to be clear about the purpose of these tests and recognize that the choice of undertaking many of them is based on individual, subjective values or preferences [50]. As such, standards of practice used to judge the severity in other health conditions are not required as a gate-keeping measure [51, 52]. In the absence of harm, access to non-invasive forms of prenatal testing should be tailored to the needs of parents [49, 51]. Healthcare providers also need to respect the boundaries set by their patients who do not wish to know any genetic information [53]. Prenatal tests labelled clearly as ‘genetic testing’ such as NIPT were easily identified by participants, allowing them to make clear decisions. However, some were blindsided by other methods of screening fetal health, such as ultrasounds, not realizing these techniques would provide similar information. They were subsequently confronted with complex decisions that they did not wish to actively make. Healthcare providers should clarify actions that constitute prenatal testing or screening, communicate similarities between the types of information provided by different tests, and the degree to which participants wish to medically assess fetal development [52]. Patient boundaries should then be respectfully observed. For example, couples agreeing to an ultrasound to visualize and ‘connect’ with their baby but who chose not to partake in prenatal screening should not have the nuchal fold measured without their expressed consent.

In line with previous research, we claim that standardization is required however in the provision of information [54, 55]. Many participants who were self-assessed to be knowledgeable and confident in their understanding of genetics, prenatal screening, testing, and NIPT did not get further counselling by their healthcare providers, especially when the test was suggested of their own initiative. This misses the opportunity to clarify misconceptions as many participants were mistakenly reassured by the test even when the results did not address their concerns [56]. It was concerning that many were informed of the test through marketing brochures from commercial test providers, raising issues of conflict of interests in patient education [57–62]. Having standardized safeguards in place would ensure the informed consent process is respected [63]. One approach may be to identify and standardize critical components of the prenatal testing, such as the procedure, the analysis, and the interpretation of results, and ensure each component is addressed – whether by the explanation of the healthcare provider to the patient, or the reverse-explanation of patient to healthcare provider. This enables healthcare providers to affirm the patient’s knowledge, bridge knowledge gaps and correct misconceptions. Concerns raised by both doctors and patients about service exigencies limiting contact time with patients also need to be addressed to enable effective and adequate informed consent [64–66].

The need for certainty both on the part of parents and gynaecologists [15–18, 18, 20, 23–26, 67] has played a large role in driving the technological development of prenatal testing [68–70], and in the promotion of NIPT as an improvement over other prenatal screening measures [71–74]. Our analysis reveals that the concept of certainty in relation to prenatal testing and screening contemplated by mothers is much broader than test accuracy. When contemplating prenatal testing and screening, mothers hypothesize possible outcomes, potential care needs, the stability of support systems, the limitations of testing, and the uncertainties inherent in a ‘healthy’ pregnancy and life. Participants recognized the transformative nature of pregnancy, acknowledging the unpredictability of their values and preferences. Given the unpredictability of these scenarios, it would be beneficial if women were given tools to cope with uncertainty. It would also be helpful for women to have access to a skilled professional to consult during the contemplative process. For example, in trying to behave in an ethical manner, many women make quality of life judgements on their prospectively disabled child by projecting themselves into the disabled child’s position. Discussing these scenarios with a trained professional could help them consider their prospective child independent of themselves by realizing that the impact and value of a disability on a person born disabled is different from that of an able-bodied person who later loses a bodily function [75]. Lastly, women emphasized the need for emotional support by their healthcare providers. They recognized that pregnancy was a period of insecurity and vulnerability and sometimes felt pressured to undertake prenatal tests. Further research could address the specific issues of insecurity and vulnerability, possibly from an intercultural comparative perspective. Women also highlighted their concerns about acting on emotion, with many pre-meditating their response to challenging decisions. Instead of suppressing their emotions, it would be valuable if women could be facilitated in harnessing their emotional responses to make meaningful decisions. Finally, a list of key actionable points on key issues emerging from our interviews and based on the qualitative analysis of the results is provided in Table 1 (Table 1).

**Table 1:** Key Actionable Points. The table contains actionable points for addressing issues related to prenatal testing and pregnancy experience. The first column identifies the issues, while the second column suggests practicable actions. The third column outlines the possible limitations of the suggested actions and their global applicability

Key Actionable Points		
Issue	Practicable action	Possible limitations
Recognize the transformative nature of pregnancy experience	• Clarify values over time • Take social and emotional aspects of decision-making into consideration • Support pregnant women in dealing with risk and uncertainty. Ideally, this would be supported by a trained psychologist/specialized nursing staff	• Demographic- specific • Feasibility may vary with local resource limitations
Standardize information provision	• Identify and standardize critical components of prenatal testing informational needs, and ensure each component is addressed by healthcare professionals • State clearly and unequivocally actions that are part of prenatal testing protocols (e.g., ultrasounds/blood tests) • Provide qualitative information of conditions screened for with prenatal testing to meet the informational needs of pregnant women • Affirm and/or clarify patient knowledge	• Issues of health literacy and functional literacy of the population in question need to be taken into consideration
Personalize care provision	• Recognize value/preference based decisions versus evidence-based decisions • Respect patient preferences/boundaries, including the preference to not undergo testing	• Resource limitations may curtail parent preferences – e.g., the use of ultrasound for bonding as opposed to medical need in resource-scarce areas • Potential issues of fairness (accessibility of private testing to those who can afford it when not assessed to be high-risk as opposed to those who cannot)

### Study limitations

This was a nationwide study, and care was taken to include migrants and underrepresented minorities. At the same time, there are significant cultural differences even among the Swiss population, especially between the different language regions. It would be worthwhile to explore the needs of women in specific cultural groups in depth for a more nuanced and differentiated approach to the prenatal counselling process, as women may raise culturally-specific needs and concerns [49].

Unlike many other countries, the Swiss population is insured through a mandatory basic insurance scheme. This may limit the applicability of our findings, especially test uptake, to other countries where the financial affordability of healthcare is a greater concern.

In spite of the best efforts of the research team and our collaborators, it was particularly challenging to recruit women who had intentionally terminated their pregnancy (*n* = 1). It would have been valuable to have a broader perspective from women who had partaken in prenatal testing and chosen to terminate their pregnancy on that basis.

Finally, the interviews for our study were conducted between 2018 and 2019, and thus the results of this study might no longer fully reflect the landscape of prenatal testing in Switzerland, including how information is provided to expecting mothers, and how prenatal testing and screening information is perceived by women.

## Conclusion

Our study highlights how women construct their understanding of prenatal genetic testing and screening, illuminates their key take-aways from the prenatal counseling process, and provides insight into further improving the clinical interaction and the process of informed consent. Healthcare providers should be mindful of the technical aspects of prenatal testing and screening that should be communicated and should also consider supporting women in complementary decision-making skills. It is worth considering how to prepare healthcare providers to provide this support and facilitate the necessary counselling process(es) given the resource limitations of the clinical setting.

### Supplementary Information

**Additional file 1.** 

**Additional file 2.** 

## Data Availability

All extra data, raw data, and materials concerning the interviews can be requested by contacting the lead author, Nikola Biller-Andorno, at biller-andorno@ibme.uzh.ch. The interview used in the study was developed for this study and was not previously published.
