# Supplementary material for: Women’s experiences with non-invasive prenatal testing in Switzerland: a qualitative analysis
Source: BMC Med Ethics. 2023 Oct 23;24:85. doi: 10.1186/s12910-023-00964-3 (PMC10594794; doi:10.1186/s12910-023-00964-3)
Supplement: Supplementary file 2 — Additional file 2. [file 12910_2023_964_MOESM2_ESM.docx]

**Supplementary material 1 - Interview guide**

Question:

What do women **understand of genetic information and its implications**,

and

how do they **balance using this information** with their **own moral/ethical stance** on childbearing in their reproductive **decision-making**?

Goals:

1. Values and needs clarification among pregnant women (with respect to their conceptions of the responsibilities of parents towards the future of their child)
2. Understand the vulnerabilities and external influences affecting women
3. Use (1) and (2) to understand:
   1. How to support women such that they can make decisions best reflective of their own values
   2. How NIPT could be further developed based on their needs and values
   3. How genetic testing in the form of NIPT should be distributed (if at all) if publically supported in Switzerland

Domains that need to be explored (used primarily to guide analysis):

1. How do women construct their **understanding** of genetics?
2. What factors **influence** how they **weigh** factors in decision-making?
3. How do they **integrate** their knowledge and understanding with their moral stance?
4. What kinds of issues do they think about when determining their **responsibilities** as parents to their future child?
5. What should or should not be controlled?

**Introduction:**

Hi, my name is ___________. I am a researcher with the Institute of Biomedical Ethics at the University of Zurich. I am interested in understanding women’s pregnancy experiences. My research is focused on a blood test some mothers do that can tell the baby’s DNA. You may know this test as the Panorama test, Praena or Harmony test.

The aim of my research is to understand the needs of pregnant women, so that we can make a recommendation about how to use this test. Some of my questions may not be directly about the test itself, but are important to my understanding.

Would you like to share your experience of pregnancy with me? *(Proceed with process of consent-taking)*

If at any time you feel uncomfortable, or if the question is one you would rather not answer, please let me know and we can move on to other questions. You can also let me know if you would like to stop the interview at any time.

**Opening Question:**

**Before we speak about your present pregnancy, could you please tell me about any other pregnancies you might have had?**

Follow with main narrative question:

**Could you tell me the story of your pregnancy? Please start from the very beginning of your pregnancy journey.**

**Expect narrative here**

*Prompts:*

- Was your pregnancy planned? How did being pregnant make you feel?
- Did you have any ideas of what pregnancy would be like? What challenges or joys were you expecting?

**I understand some women have had previous miscarriages or abortions, that has an impact on how they view their subsequent pregnancies. Have you had any such experiences?**

Lead-in to semi-structured questions: **Thank you for sharing your pregnancy experience with me. I would like to ask you some questions now about how you envisioned your role as a parent, and later about your experiences with your medical visits during the pregnancy.**

| Questions | Remarks (for researcher’s background knowledge; not formal questions for participants) |
| --- | --- |
| **Values, parental responsibility and community support**  What would you imagine is your role as a parent?  What kind of experiences do you hope to have together with your child?  Do you have any aspirations for your children?  What did you see as your responsibilities towards your child during pregnancy?  What actions did you feel were the most important to having a healthy child?  What does health mean to you?  Was there anything that you felt pregnant women were often advised but was unnecessary to do?  Were there any expectations that you felt from your family or friends about the child that you were about to have?  Who did you involve in decisions about your pregnancy? How did you involve them?  Were there any social policies that you took into consideration with your pregnancy?  Were there any specific insurance considerations that you thought to check on when you got pregnant?  How did you imagine the community around you would help with your child?  To which community do you feel you belong?  Did you feel that there were expectations of how your child should be before you received help from your community?  Did you feel like you had to behave in a certain way during your pregnancy before people would help you?  What was the experience of announcing your pregnancy like?  **Prenatal Visits**  *Not all women in the study may have had antenatal care. If this information has not yet been volunteered, start by asking:*  Have you had any visits to your doctor? What prompted these visits?  *If it was known they had received prenatal care, preface instead with:*  So you mentioned earlier that you visited a doctor (or midwife) when you were pregnant..  *Followed by:*  **Could you tell me about your doctor’s (midwife’s) visits during your pregnancy?**  **What kind of things did you discuss with your doctor (midwife)**?  Follow-up questions:   - Was there anything you particularly looked forward to during these visits? - Were there any difficult experiences for you? - What was your experience of prenatal testing like? - Did you feel like you were kept well-informed of the tests you had done and their results, or did you feel left in the dark? - Were you aware of any forms of genetic testing during your pregnancy? *(explore what is known about NIPT and amniocentesis/CVS)* - Were you already keen on performing a genetic test on your baby, even before your doctor offered it? - Did your doctor or midwife tell you anything about genetic testing in particular? - Was there any information you sought out independently or asked friends and family about genetic testing (or NIPT)? (for example, something that you remembered doing a google search or visiting a pregnancy forum to find out) - Was genetic testing (or NIPT) different from the other prenatal tests to you? - What was your experience of genetic testing like?   - What did you know about NIPT?   - Why did you choose to/choose not to use NIPT?   - Were there any specific insurance considerations that influenced your decisions about genetic testing?   - How did you decide what kind of disease to test for?   - What did your doctor discuss with you when you spoke about NIPT? - What do you know about the conditions presently tested for?   - Why are these conditions being tested?   - Are these important to you?   - What do you know about Down’s Syndrome? How did you come to this understanding?   - Were there other conditions you would have liked to know about?   - Are you aware of these conditions in the society around you? - How were you informed of the test results? What did you understand of it? - What did you plan to do after knowing the results? - What is your take on aborting an affected pregnancy? - Did you feel confident about the decisions you made at the time? - Was the test helpful to parental anxiety or added to it? - What do you think it would be like, if prenatal tests were not available, and you only found out the characteristics of your baby for the first time at birth? - NIPT presently screens for only a few specific conditions. Scientists are trying to make it such that it will be able to look at all the baby’s genes, so that as many diseases as possible can be screened for.   - Is this something that you would want to do?   - (If yes:) Some geneticists say that all of us have some abnormalities in our DNA, but they do not always affect us in harmful ways. How our genes affect us is unfortunately not always predictable. How then would you decide which abnormalities found are important to you? - Selecting or creating the best possible child – what does that mean to you as a parent? - How would that translate in your actions? *(Explore themes of* ***abortion*** *and* ***gene-editing****)* - Is there something you might not want to know about your child’s genes? - Some people feel pregnancy is a normal process of the body, and does not need medical guidance. Other people prefer to have a doctor or midwife oversee their pregnancy. What do you think of pregnancy? - Some tests are important during the pregnancy, but some others are thought as unnecessary medical involvement. Do you see genetic testing as unnecessarily medicalizing pregnancy? - What do you think is the responsibility of parents to a test like NIPT? - If you find out about a genetic condition your child may have, for example your child may have genes that make it more likely to have heart disease, do you then consider your child as having heart disease?   **Decision-making**  How would you like your doctor to involve you in decisions about NIPT?  What was the most helpful guidance you received from your doctor? What was the least helpful? (If not about NIPT, then ALSO ask: what about with regard to NIPT?)  What kind of environments make you feel supported in making decisions in line with your values? (OR: What would have helped you make decisions in line with your values during difficult situations? *(specify this if it comes up in the course of the conversation))*  Were there any particular people who were important to you to involve in this decision?  What do you think of childhood vaccinations?  How do you feel about parents who choose not to vaccinate their children?  Do you think parents have a duty to society by choosing not to have children with genetic diseases?  Do you think, if genetic selection is normalized, there would be less support for choosing a child with a preventable medical condition?  If a couple chooses to have a child whom they know will have a genetic abnormality, should insurance cover this child’s future medical cost, or should the parents bear full responsibility?  Do you think insurance should provide all women with access to NIPT?  And if NIPT is covered by insurance, do you think they should also have access to the child’s genetic data? Who should have access to this data? | What kinds of issues do they think about when determining their **responsibilities** as parents to their future child?  What kind of life do they imagine for their child?  What kind of life is acceptable to them?  ** what parents describe is important to them relates then to the purpose of prenatal tests and therefore also to the relevance of/nature of genetic testing (i.e. whether genetic information is important, and if so, what kind of information)  What are the different levels of social affectations –   - - - Personal     - Interpersonal (spousal, relations, friends)     - Community (work, society, religion),     - Support (government, insurance)   Who are the stakeholders of the (future) child – and to what extent should stake-holders (or in what discrete aspects should stake-holders) have any say/should their values be considered?  What kind of support do women perceive they would receive as parents?  What kind of expectations hinge on receiving such support?  How does this affect the prospective/speculative role envisioned by parents of their child in society? And how does it affect their decision-making with respect to selecting children?  What kinds of external influences affect perspectives, and how?  The reservations parents have when sharing details about their pregnancy can be reflective of both expectations of society and of parents towards their child – parental assessment of social solidarity   - Expect some remarks on blood tests/US/testing, and their experience of it - Needs   Possible themes and probes:   - How and when did they learn about genetics? - Do they know NIPT is a genetic test? - What is genetic testing? - Who does the results of this testing apply to? - What other implications might it have? - Advice from doctors/midwives - How much (how in depth) and what do parents think is important to know of genetic tests? - Sources of information - Of all the genetic diseases, how did they choose to test for specific ones? If all genetic diseases were available to screen for, what then would they test for? - Experiential knowledge (either self or other) - Examples in the media/community - Follow-up actions - Thinking about consequences - How secure do women feel about making decisions on the future of their pregnancy based on these test results? - How would it be different for women if they had met their child with a disability for the first time at birth? Would they be more accepting or less so?   What constitutes over-medicalization?  Where is the locus of control in pregnancy – who should hold these reins?   - Do parents perceive a difference between what is tested for, and how it may or may not be expressed in the child? Or how it may be expressed in different (and unpredictable) degrees? - What does health mean in the context of genetics?   Who has access to their child’s genetic data?  What would they do with the results? |

**Post-pregnancy**

a. What would you do the same in your next pregnancy (and why)?

b. What would you do differently in their next pregnancy (and why)?

c. Is there any advice you would like to give to doctor’s and nurses, based on your experience of your pregnancy? How about with regards to your experience of NIPT?

d. Is there anything else you would like to share before we close?
